# Health Care Disparities Between Men and Women With Type 2 Diabetes

**DOI:** 10.5888/pcd15.170120

**Published:** 2018-04-19

**Authors:** Marady Sabiaga Mesa

## Abstract

**Introduction:**

Regular medical checkups indicate a patient’s level of adherence to health care treatment, and the frequency of cancelled appointments or no-shows can indicate adherence. This study investigated the use of health care services by men and women and its impact on the control of their type 2 diabetes.

**Methods:**

This study observed 100 patients with type 2 diabetes aged 45 years or older who lived in Ventura County, California, during January 1, 2015, to January 31, 2016. The data were collected by Magnolia Family Medical Center. A Pearson χ^2^ test compared differences between men and women in whether they received a glycated hemoglobin A_1c_ (HbA_1c_) test in previous 6 months, a low-density lipoprotein cholesterol test in previous year, and a retinal examination in previous year. A Wilcoxon signed-rank test compared attendance to medical appointments and HbA_1c_ values for men and women.

**Results:**

Women had a higher rate of scheduling, cancelling or rescheduling, and showing up to their medical appointments than did men, and men had a higher median HbA_1c_ value than did women; all the Wilcoxon signed-rank tests showed a significant difference (*P* < .001). None of the χ^2^ tests were significant.

**Conclusion:**

Although men and women had similar health care services for diabetes, men had less control of their disease and took less advantage of medical appointments than did women.

## Introduction

The prevalence of type 2 diabetes increased from 1980 through 2014 (1). Dieting, exercising, attending regular medical check-ups, and screenings may prevent or control such disease (2). Regular medical checkups indicate a patient’s level of adherence to health care treatment, and the frequency of cancelled appointments or no-shows can indicate adherence. Several screenings, such as retinal examinations and laboratory work for glycated hemoglobin A_1c_ (HbA_1c_) and low-density lipoprotein (LDL) cholesterol, are recommended for proper diabetes care and disease prevention (3).

HbA_1c_ measurements are used to observe the patient’s blood glucose level. The higher the HbA_1c_, the more sugar is found attached to the red blood cells; HbA_1c_ should be less than 5.7% (3). People with diabetes have an HbA_1c_ of 6.5% or higher (3). LDL cholesterol is a measurement of low-density lipid to determine the risk of developing heart disease. Patients are at a higher risk of heart diseases if they have diabetes and have high levels of LDL cholesterol (3). A retinal examination, or a funduscopy, checks for eye diseases. Uncontrolled diabetes can lead to diabetic retinopathy (3). According to American Diabetes Association’s *Standards of Medical Care in Diabetes*, HbA_1c_ measurements should be done at least once every 6 months, LDL cholesterol measurements should be done at least once every 5 years, and retinal examinations should be done at least once every 2 years (3). If patients are taking statins to lower blood pressure, the frequency of LDL cholesterol measurements depends on the physician and patient (3). Patients with any levels of diabetic retinopathy should have retinal examinations at least once every year (3).

Proper treatments are done after an individual has had diabetes diagnosed. Preventing or slowing the progression of such disease depends ultimately on the patient. This is a health issue because a disease can progress without early detection, proper diagnosis, treatment, and full commitment of the patient.

Several factors in a person’s life can create difficulties in diabetes prevention and control, including the level of adherence to recommended schedules of medical care services. Shalev et al and Krämer et al have found significant difference between men and women and their use of medical care (4,5). However, both studies were generalizable to individuals outside of the United States. Vaidya et al found that women used preventive care more frequently (6); however, they did not observe patients already diagnosed with diabetes. Bertakis et al found that women used health care services more often than did men (7). However, that study examined data on all health care services, including those that may not pertain to men.

The objective of my study was to determine whether differences exist between men and women in the control of diabetes and the use of medical appointments. 

## Methods

The study cohort was patients with type 2 diabetes aged 45 years or older who lived in Ventura County, California, and were regularly checked for diabetes care at Magnolia Family Medical Center. I obtained the data from Magnolia Family Medical Center with the approval of the medical director. The Quality Improvement and Research: Spreading Effective and Efficient Diabetes Care (QIR/SEED) department of Magnolia Family Medical Center collected data from the clinic’s electronic health records system, Cerner (Cerner Corporation), through Cerner’s Explorer Menu application. The Explorer Menu application produced a report of patients with a Systematized Nomenclature of Medicine–Clinical Terms (SNOMED–CT) problem code of 197763012, which was a diagnostic code for diabetes mellitus 2 in Cerner. The application was then used to identify all patients with that SNOMED-CT code who were aged 45 years or older and who came into the clinic with an appointment during January 1, 2015, to January 31, 2016. The report included data on patient demographics, diagnoses, history, primary care provider name, and appointments.

With the report generated by the Explorer Menu, QIR/SEED collected data on patients who had diagnoses of hypertension or hyperlipidemia and who did not have anemia. QIR/SEED screened out patients who were not regular patients of Magnolia Family Medical Center and who were seen only for a nonprovider appointment. Because of the time involved in gathering information for each patient, the first 50 men and 50 women who fit the criteria from a stratified random sample were included in the study. The study focused on the 100 patients’ medical activities from January 1, 2015, to January 31, 2016.

Demographic variables analyzed were age (45–54, 55–64, and ≥65 years), race/ethnicity (Asian, black/African American, other or more than 1 race, white Hispanic, and white non-Hispanic), and sex. The racial/ethnic distribution of this sample was compared with that of Ventura County, which is 84.5% white Hispanic and non-Hispanic (8). Patient appointment data analyzed were the number of no-shows, number of cancelled or rescheduled appointments, and total number of appointments. Show-up rates were calculated by subtracting the number of no-shows from the number of total appointments. Laboratory data for HbA_1c_ and LDL cholesterol were reviewed and noted as to whether they were outdated, up to date, or not done. Retinal examination status was noted as to whether the examinations were outdated, up to date, could not be performed, or the patient had never had one. If the patient did not get their HbA_1c_ test done within 6 months of their last HbA_1c_ test during the study period, their HbA_1c_ status was recorded as outdated. Similarly, retinal examinations and LDL cholesterol tests that were not done within 1 year from the last examination during the study period were recorded as outdated. The number of canceled and rescheduled appointments were recorded to observe the patients’ commitment to medical appointments concerning diabetes. The number of no-shows is the number of times a patient had an appointment and failed to show up. The total number of appointments scheduled included no-shows and kept appointments during the study’s timeframe.

Patients’ names, addresses, medical record numbers, date of birth, and any identifying factors were excluded from the data analyzed. Medical record numbers were changed to a random value from 1 to 100 to protect the patients’ identities. Factors such as insurance coverage, transportation, jobs, and family commitments were not considered in the study because they are extrinsic factors. Also not recorded was time since a patient received a diagnosis of diabetes. Medication adherence was measured through the patients’ verbal responses to their physician’s questions about whether or not they were taking their medications; to avoid the limitations associated with self-reported data, data on medication adherence were excluded from the study. A letter of exemption from National University’s institutional review board was obtained to investigate these data.

RStudio (RStudio) was used to analyze and interpret the data. In RStudio, box plots were produced to check for outliers and visualization of any possible differences. The box plots were also used for analyzing the distribution of the data set. A Pearson χ^2^ test compared differences between men and women in whether they received an HbA_1c_ test in previous 6 months, an LDL cholesterol test in previous year, and a retinal examination in previous year. The χ^2^ test was also used to examine whether these variables were dependent on each other. A Wilcoxon signed-rank test was performed on sex versus total appointments scheduled, appointments cancelled or rescheduled, rate of showing up, and HbA_1c_ values. The Wilcoxon signed-rank test was also used to observe any differences between the medians for men and women. The level of significance used for both the χ^2^ test and Wilcoxon signed-rank test was α = .05.

## Results

Of 100 patients in this study, 7 were Asian, 2 were black/African American, 45 were white non-Hispanic, 32 were white Hispanic, and 3 were other or more than 1 race. Only data on the white non-Hispanic and white Hispanic groups were analyzed because the other 3 groups had small numbers. This racial/ethnic distribution is similar to that of the Ventura County population. Eighty-eight percent of the white non-Hispanic group had an outdated HbA_1c_ test, 45.1% had an outdated LDL cholesterol test, and 66% had an outdated retinal examination. In the white Hispanic group, 86.5% had an outdated HbA_1c_ test, 56.8% had an outdated LDL cholesterol test, and 68.6% had an outdated retinal examination.

Of the 100 patients, 36% were aged 45 to 54 years (21 men and 15 women), 44% were aged 55 to 64 years (23 men and 21 women), and 20% were aged 65 years or older (6 men and 14 women). The range for HbA_1c_ values for women was 5.2 to 12, with an outlier of 12. The range for HbA_1c_ values for men was 5.8 to 12, with no outliers. The range of total appointments for women was 15 to 118 and for men was 6 to 58; for women, 118 was an outlier, and for men 58 was an outlier. The range of values for showing up to an appointment for women was 16 to 116 and for men was 6 to 58; for women, 116 was an outlier, and for men 58 was an outlier. The range of values for cancelled or rescheduled appointments for women was 5 to 57 and for men was 3 to 19; 57 was an outlier for women, and there was no outlier for men.

During January 1, 2015, to January 31, 2016, most men (76%) and most women (70%) had had at least 1 HbA_1c_ test done within 6 months (Table). HbA_1c_ tests were outdated for 18% of men and 30% of women. Most men (90%) and most women (84%) had had an LDL cholesterol test within the previous 6 months; 8% of women and 10% of men had an outdated LDL cholesterol test. At least 1 retinal examination had been recorded in the past year for 62% of men and 56% of women; 18% of the men and 16% of the women had not had a retinal examination in the past year. Sixteen percent of men and 26% of women had an outdated retinal examination. No significant associations were found between sex and whether or not patients received any of these services within the designated time frame.

**Table:** Use of Health Care Services Among 100 Patients With Diabetes Aged 45 Years or Older Regularly Seen at Magnolia Family Medical Center, Ventura County, California, January 1, 2015, to January 31, 2016

Variable	Population	Sex		*P* Value^a^
		Male	Female	
**HbA_1c_ value, median**	7.2	7.4	6.8	<.001^a^
**Total no. of appointments, median**	21.5	16.0	25.5	<.001^a^
**No. of appointments showed up for, median**	18.5	14.0	23.5	<.001^a^
**No. of cancelled or rescheduled appointments, median**	7.0	6.0	11.5	<.001^a^
**Had HbA_1c_ test within previous 6 months, n (%)**				
Yes	73 (73)	38 (76)	35 (70)	.99^b^
Not done	3 (3)	3 (6)	0	
No	24 (24)	9 (18)	15 (30)	
**Had low-density lipoprotein cholesterol test within previous year, n (%)**				
Yes	87 (87)	42 (84)	45 (90)	.54^b^
Not done	4 (4)	3 (6)	1 (2)	
No	9 (9)	5 (10)	4 (8)	
**Had retinal examination within previous year, n (%)**				
Yes	59 (56)	31 (62)	28 (56)	.63^b^
Not done	17 (17)	9 (18)	8 (16)	
Not applicable^c^	3 (3)	2 (4)	1 (2)	
No	21 (21)	8 (16)	13 (26)	

Abbreviation: HbA_1c_, glycated hemoglobin A_1c_.

a Based on Wilcoxon signed-rank test where α = .05.

b Based on Pearson χ^2^ test of association where α = .05.

c Patients not able to obtain a retinal examination because of blindness or surgery (which would mean the patient’s care was being handled by an ophthalmologist and the patient would most likely have received a retinal examination).

Men had a higher HbA_1c_ median than did women (Table). The median of appointments that men showed up for was 14.0, while for women the median was 23.5 (*P* < .001). Women had a higher median of cancelled or rescheduled appointments than men did (*P* < .001) and a higher median number of total appointment than men did (*P* < .001). Therefore, differences between use of appointments by men and women and their median HbA_1c_ values were significant (Table).

## Discussion

This study found a difference in the control of diabetes as well as the use of medical appointments between men and women. Similar results were observed in studies by Bertakis et al, Legato et al, Grant et al, and Singh-Manoux et al (7,9–11). Each study suggested a difference between the prevalence of diseases, including diabetes, between men and women. Comparable to the findings of Shalev et al, the results of this study also found that women had more scheduled appointments than did men (4).

Men and women at Magnolia Family Medical Center were provided similar health care services and recommendations; such services included getting retinal examinations, complying with schedules for receiving laboratory tests, and showing up to their medical appointments. However, women had better control of their blood glucose levels. Thus, making sure both sex groups had up-to-date blood work and retinal examinations did not guarantee that both sex groups had similar diabetes control.

My study has a few strengths. For instance, the study solely focused on a population with a medical condition; thus, the study was specific. I did not collect the data; hence, no researcher-generated data-collection biases could affect the outcome. The study also had a long time frame of 1 year. Data were not collected from surveys, but rather through physician documents, laboratory reports, retinal examination reports, and scheduling reports. Thus, no biases could result from patient self-report or me.

This study also has limitations. The data collected were from a clinic; therefore, some outliers were found. Clinic providers had different data entry techniques; thus, some data may not have been collected. Because the data were collected through a computerized system that generated reports entered by people, data entry errors and other human errors limit the accuracy of the data. The study did not include data on the length of time that patients had had a diabetes diagnosis, and the findings are pertinent only to the population of patients with diabetes at Magnolia Family Medical Center. Another limitation was the population size. The study examined data only for patients with type 2 diabetes who had hypertension or hyperlipidemia and who were taking similar medications. The study focused only on patients regularly seen by their primary care provider in Magnolia Family Medical Center. A bigger population size should be considered for future studies. The study was also biased toward recording appointments made with Magnolia Family Medical Center only. Other clinic appointments should be recorded for future studies.

Conclusions drawn from this observation are generalizable only to the population in the study. This study solely observed individuals with type 2 diabetes and focused on the population with diabetes at 1 clinic in Ventura County, California. The observations did not show an association between regular checkups and a decreased gap between proper diabetes care in both sex groups. Although the medical treatments of the men did not differ from those of the women, men had less control of their disease; thus, sex-specific medical treatments and health education should be investigated. Moreover, when treating men with type 2 diabetes, a care provider and health professional must stress the importance of controlling blood glucose levels and health care utilization. Further studies should also investigate what causes men to have less control of their blood glucose levels. For a generalizable study, factors such as medication adherence, types of insurance and coverage, the length of time since type 2 diabetes was diagnosed, age at which type 2 diabetes was diagnosed, and race/ethnicity should be included. Other extrinsic factors should be included because they may influence behaviors related to keeping appointments and compliance with medical treatments.

Overall, men were found to have lower rates of cancelling or rescheduling a medical appointment; however, they also had a lower rate of showing up to their appointments. Regardless of men and women having similar rates of getting their blood work and screening for retinal examinations, men were still found to have a significantly higher HbA_1c_ median compared with women. Therefore, even when both sex groups were provided similar health care services for diabetes, men still had less control of their diabetes. This study will contribute to improving care for diabetes patients and will encourage care managers to work closely with their patients.
